# Bridging the Gap Between Patient Experience and System‐Level Review: A Call for Discipline‐Specific Communication in Hospital‐to‐Home Transitions

**DOI:** 10.1111/hex.70748

**Published:** 2026-06-26

**Authors:** Qian Yang, Juanjin Wang, Peihua Xiao, Huan Du

**Affiliations:** ^1^ Department of Rehabilitation Medicine Suzhou Ninth People's Hospital Suzhou China; ^2^ Department of Nursing Suzhou Ninth People's Hospital Suzhou China

Dear Editor,

We read with great interest the qualitative study by van Grootel et al. [1] and the scoping review by Thompson et al. [2], both published in Health Expectations. The former captures the lived experiences of patients during hospital‐to‐home transitions, revealing the critical challenge of ‘information recall and processing’, patients often feel overwhelmed by information provided at the wrong moment and in an undifferentiated manner [1]. The latter systematically confirms that ‘clinician–patient communication’ and ‘experiences of discharge’ are key determinants of care quality for people with multiple long‐term conditions (MLTC) [2]. We commend these important contributions for bringing patient voices and system‐level evidence to the forefront of transitional care research and wish to extend the discussion by highlighting an underexplored dimension: discipline‐specific communication needs within the allied health workforce.

Both studies rightly identify communication breakdowns as central to suboptimal transitions. Van Grootel et al. [1] found that patients struggled to retain and act upon discharge information, particularly when multiple professionals provided instructions that were not well coordinated. Thompson et al. [2] concluded that poor communication between specialists, conflicting advice and rushed discharge planning were pervasive themes across the literature. However, both studies treat ‘communication’ as an undifferentiated concept, aggregating all allied health professionals into a single category. Our clinical experience in rehabilitation medicine suggests that this aggregation masks a critical issue: communication barriers and needs vary significantly across different rehabilitation disciplines.

In our practice, we observe that speech‐language therapists, dietitians, and occupational therapists face qualitatively distinct barriers that are rarely captured in generic discharge summaries or quality indicators. For speech‐language therapists, inadequate handover of dysphagia management plans, including modified diet consistency, aspiration precautions, and specific compensatory strategies, can have immediate and serious post‐discharge consequences. A recent case in our rehabilitation unit illustrates this: a post‐stroke patient with dysphagia was discharged with only generic ‘swallow precautions’ documented in the discharge summary. The community therapist lacked details of the prescribed modified diet consistency and the specific chin‐tuck and head‐turn strategies recommended by the hospital speech‐language therapist. Consequently, the patient was inadvertently prescribed an unsafe diet consistency and positioned incorrectly during meals, resulting in aspiration pneumonia and unplanned readmission within 1 week [3]. Such discipline‐specific information is often lost or overly generalised, leading to avoidable adverse events.

Dietitians face similar challenges. Nutrition care plans, including energy and protein requirements, texture modifications and supplementation schedules, are frequently omitted from discharge summaries or presented in formats that community providers find difficult to implement [4]. In our setting, we have observed that when a patient with diabetes and chronic kidney disease is discharged without a clear, dietitian‐signed plan for carbohydrate counting and potassium restriction, community providers often default to generic dietary advice, leading to glycemic instability and electrolyte imbalances. Occupational therapists, who assess home safety, adaptive equipment needs, and activities of daily living, often find their recommendations overlooked when discharge planning is driven primarily by medical or nursing priorities. Hellman and Lindström [5] documented how occupational therapists’ home modification and assistive device recommendations are frequently deprioritized, resulting in delayed or unsafe home discharges.

This pattern is not uniform. Physical therapists’ assessments, by contrast, appear more consistently integrated into discharge decisions, a pattern that Thompson et al. [2] indirectly noted when they reported that patients valued ‘consistency between care providers’. In our observation, physical therapists’ mobility assessments and discharge recommendations (e.g., walking aids, home exercise programs) are almost always documented and actioned, likely because their role in determining discharge readiness (e.g., safe mobility) is well understood by medical and nursing colleagues. This disparity suggests that the visibility and perceived urgency of a discipline's contribution influence whether its information is reliably transmitted across care settings.

We propose that transitional care interventions must move beyond generic ‘communication’ strategies and adopt discipline‐specific approaches. Three actionable, low‐cost steps are:

1. Discipline‐specific checkboxes in discharge summaries for speech‐language therapy (e.g., modified diet grade, aspiration risk status, safe swallowing strategies), dietetics (e.g., nutrition care plan, supplement regimen), and occupational therapy (e.g., home safety modifications, assistive device needs). These checkboxes would prompt hospital teams to include information that is currently often omitted.
2. Brief verbal handover protocols for high‐risk disciplines such as speech‐language therapy. A 5‐min telephone call from the hospital speech‐language therapist to the community counterpart, highlighting key dysphagia management parameters, could prevent aspiration events. Similar protocols could be developed for dietitians when discharging malnourished or metabolically complex patients.
3. Structured feedback loops from community providers to hospital teams. Community therapists should be able to report when received information is incomplete or unactionable, enabling hospital teams to improve their handover practices. This could be implemented as a simple online form or a quarterly quality improvement review.

Future research should systematically compare handover completeness and post‐discharge adverse events across allied health disciplines. Such studies could use audits of discharge summaries (checking for discipline‐specific elements) linked to patient outcomes (readmission rates, functional recovery). Qualitative studies could explore why some disciplines’ recommendations are more reliably transmitted than others, and whether interventions such as the checkboxes proposed above reduce disparities.

Without this granularity, as van Grootel et al. [1] and Thompson et al. [2] have compellingly shown, we will continue to overlook a key driver of fragmented care. We hope that integrating discipline‐specific insights into the frameworks developed by these authors, and ideally through collaborative efforts between researchers and rehabilitation practitioners, may further strengthen the design of transitional care interventions and ultimately improve outcomes for patients with complex rehabilitation needs.

## Author Contributions

**Qian Yang:** conceptualisation, writing – original draft, writing – review and editing. **Juanjin Wang:** conceptualisation, writing – review and editing. **Peihua Xiao:** supervision, validation. **Huan Du:** conceptualisation, writing – review and editing, supervision.

## Funding

The authors have nothing to report.

## Ethics Statement

Ethics approval was not required for this Letter as it does not involve human participants or animal studies.

## Consent

Patient consent is not applicable as this manuscript does not report any primary data from human participants.

## Conflicts of Interest

The authors declare no conflicts of interest.

## Data Availability

Data sharing is not applicable to this article as no new data were created or analysed.
